# The Localization, development and validation of a survey instrument to assess attitudes toward professionalism based on the opinions of clinical teachers

**DOI:** 10.1186/s12909-022-03987-3

**Published:** 2022-12-29

**Authors:** Adeleh Hosseinizadeh, Mahsa Shakour, Leila Afshar

**Affiliations:** 1 Department of Medical Education, School of Medical Education, Shahid Baheshti University of Medical Sciences, Tehran, Iran; 2 Arak University of Medical Sciences, Arak, Iran; 3 Medical Education Department, School of Medicine, Arak University of Medical Sciences, Arak, Iran; 4 Medical Ethics Department, School of Medicine, Shahid Beheshti University of Medical Sciences, Tehran, Iran

**Keywords:** Medical education, Localization, Questionnaire, Clinical professors, Professionalism, Factor analysis

## Abstract

**Background:**

This study examined the validation of a survey instrument to assess attitudes toward professionalism based on the opinions of clinical teachers in Iran and localized it.

**Methods:**

The research is applied-descriptive, that is conducted in two stages: qualitative and quantitative. Fifteen expert professors in the field of professionalism from Iranian universities of medical sciences participated in qualitative stage by Delphi. sampling method was purposive. In the quantitative stage, using simple and quota sampling method, 82 samples were collected from all clinical professors of first ranked universities of medical sciences. We used “The Penn State College of Medicine Professionalism Questionnaire”( PSCOM) as a basic questionnaire. Its validity assessed by Delphi and survey. Some items changed, deleted and added. Then it confirmed by confirmatory factor analysis.

Data analysis was performed using one-sample t-test and SmartPLS software.

**Results:**

Seven dimensions and 48 final indicators were approved and all 7 dimensions were in good condition. Among the approved dimensions, “the enrichment” with a factor load of 0.238, was the biggest factor in measuring the variable of professionalism. The dimensions of “respect”, “responsibility”, “altruism”, “duty”, “honor, honesty and justice”, “respectively” with factor loads of 0.215, 0.212, 0.163, 0.146, 0.106 and 0.047 are ranked in order.

**Conclusion:**

the most dimensions in PSCOM are accepted by experts and teachers, but there are many differences in details. If we want to have an accurate tool for measuring attitudes toward professionalism, then, it is necessary to study localization and validation of instrument to assess attitudes toward professionalism in every new community. Delphi and factor analysis could be useful for assessing tool in new community.

## Introduction

Professionalism is the core of competency for physicians [1]. professionalism includes a set of values, behaviors, and relationships that underpins the public trust in doctors [2]. It can be considered as a set of behaviors, goals and characteristics that express a profession or a professional person. The main dimensions of professionalism include: integrity, respect, compassion, justice, professional enrichment, and accountability and responsibility [3]. A physician’s capabilities are not dependent only on his medical knowledge, clinical judgment, and skill in performing medical procedures, but also on his mental and practical belief in professionalism. Admittedly, without a solid tool to evaluate the current attitude towards professionalism, the discussion of how to teach professionalism seems useless [4]. According to Jaha et al. (2007), one of the basic aspects of medical education is related to creating a suitable attitude towards medical professionalism in students. Developing appropriate attitudes toward professionalism in students is essential because these attitudes contribute to future practice and affect physicians’ relationships with patients, the quality of care they provide, and ultimately, health and disease outcomes [5]. Despite the importance of this issue, creating and influencing the formation of professional personality and behaviors related to professionalism is one of the most challenging and problematic issues, and assessing it between medical teachers and students is difficult.

Blackall et al. (2007) [6] conducted a study entitled Professionalism in Medical Education: the development and validation of a survey instrument to assess attitudes toward professionalism. They assessed attitudes toward professionalism in an academic medical center. The paper described the development and factorial validity of an instrument to measure attitudes toward professionalism in medical education. Respondents were asked to determine how much they agree with each of the American Internal Medicine Board’s identifiable factors of professionalism, which include accountability, altruism, duty, enrichment, equity, honor and integrity, and respect. Using a factor analysis of the intercorrelations of responses seven factors with a specific value of more than one was identified. These factors included accountability, altruism, duty, enrichment, equity, honor and integrity, and respect. They expressed that six of the identified items have concepts similarity with the original items and only the dimension of justice was identified as a new dimension in this study [7].

Jauregui (2016) conducted a study entitled “Emergency Medicine Resident Perceptions of Medical Professionalism”. The survey was conducted between graduating residents and developed using the American Board of Internal Medicine’s "Project Professionalism" and the Accreditation Council of Graduate Medical Education definition of professionalism competency. The results showed “altruism” domain valued significantly lower and those in the "respect for others" and "honor and integrity" valued significantly higher [8]. A study by Campbell et al. (2007) on 3504 practicing physicians in internal medicine, family practice, pediatrics, surgery, anesthesiology, and cardiology, found that the highest average was related to management of conflicts of interest [9]. In a study by Agha Mohammadi et al. by assessing the attitudes of clinicians towards professionalism, he showed that the professional attitude of medical professors towards the indicators of enrichment, honor, integrity and respect are ranked the highest and the indicators related to altruism and accountability are ranked at the lowest level [10].

The Penn State College of Medicine Professionalism Questionnaire(PSCOM**)** is one of the first valid and reliable surveys of attitudes among medical students, residents, and faculty that reflects seven elements of professionalism (accountability, altruism, duty, enrichment, equity, honor and integrity, and respect [11]. On the other hand, professionalism is specific for every society and changes by culture. Therefore the researchers in this study to assess the attitude of clinical teachers in medical schools in Iran couldn’t use it without any localization and assessing validity. The researchers used mixed methods to localize and assess validity and at the end they used confirmatory factor analysis to confirm.

## Methods

### Design and setting

Research was in two stages: 1) qualitative, Delphi and 2) quantitative, survey. Research population in this study, in the qualitative stage, was 15 expert professors in the field of professionalism from Iranian universities of medical sciences, who were selected by purposive sampling. In the quantitative stage, using simple and quota sampling method, 82 samples were collected from all clinical professors in 9 universities of medical sciences in Iran.

### Data collection and analyses

The research tool was a questionnaire. In 1994, the US Internal Board developed a preliminary questionnaire on professionalism that includes six dimensions of accountability, enrichment, integrity, altruism, accountability, and respect. This questionnaire had 36 components and each dimension included one component as a representative of each dimension and 5 other components [12]. And in 2004, researchers at the University of Pennsylvania decided to validate the tool and eventually developed the PSCOM, which has a new dimension that includes seven dimensions: accountability, enrichment, fairness, integrity, altruism, accountability, and respect. This questionnaire should have been revisited and reviewed, but the researchers stopped working and did not continue with their research [11]. At first we used Delphi to determine dimensions and their components of questionnaire. The Delphi technique is a well-established approach to answering a research question through the identification of a consensus view between experts. Participants are able to reconsider their opinion based on the anonymized opinions of others. The validity of the questionnaire was confirmed using CVI and CVR indicators and the reliability of the questionnaire was confirmed using Cronbach’s alpha coefficient which was higher than 0.7. Delphi was done in two round. Participants were 15 experts. After Delphi, 82 selected clinical teachers answered the new questionnaire. They expressed their opinion about the dimensions and their components by scoring from 1 to 5 (completely agree-completely disagree) and then graded their attitude towards those dimensions of professionalism from 1 to 5.Data analysis was performed using confirmatory factor analysis by SmartPLS software.

## Results

Demographic statistics of responders show that 40% of the participants were between 51 and 60 years old and 74% had the rank of associate professor and 70% were men and finally 74% had moderate and more familiarity.

The results of first stage is summarized in the table (1). They have been gathered to examine the attitude of experts towards the dimensions of medical science professionalism in the professional character evaluation instrument, using the survey method, by several rounds of questionnaire distributions among experts, which the results of them, are presented separately for each dimension. In stage two we gathered the attitude of clinical professors towards the dimensions of medical science professionalism in the professional character evaluation instrument, using the survey method and a one-sample t-test, which the results of, are presented separately for each dimension below: Table (2) One-sample t-test of clinical professors’ attitudes about the dimensions of professionalism.

**Table 1:** Results of the first stage for identifying dimensions of medical professionalism

		PSCOM components	The final result of two rounds of polling
Accountability	1	Collaborates respectfully and in coordination with a group to improve patient care	was approved by experts
	2	Accepts individual accountability for decisions related to patient care	From the dimension of honor and honesty was transferred to this dimension
	3	Acts dutifully to make arrangements and meet requirements	From the dimension of honor and honesty was transferred to this dimension
	4	Reacts to constructive criticism and increases its capabilities	At this stage, it was edited grammatically
	5	Recognizes their limitations in terms of professional knowledge and ability	At this stage, it was edited grammatically
	6	Committed to caring for the patient in a cost-effective manner	It was transferred from the dimension of excellence to this dimension
	7	Considers themselves responsible and accountable for the problems created for patients	According to experts, this dimension was considered as the seventh indicator
	8	Recognizes their limitations in terms of professional knowledge and ability	Transferred from this dimension to the dimension of enrichment
Enrichment	1	Demonstrates initiative and assistance in the personal and professional development of colleagues	Approved by experts
	2	Take the time to review the work of other colleagues and provide helpful and constructive comments to improve it	Approved by experts
	3	Participates in meetings, seminars and presenting student research in the faculty as a support activity	Approved by experts
	4	Looking for your personal enrichment	Approved by experts
	5	Contributes to the well-being and development of new faculty members	Approved by experts
	6	purposefully Participates in teaching the department and the medical school	Approved by experts
	7	Shows flexibility to changing priorities and transformations	From the dimension of accountability was transferred to this dimension
	8	Committed to caring for the patient in a cost-effective manner	From the dimension of accountability was transferred to this dimension
	9	Recognizes their limitations in terms of professional knowledge and ability	From the dimension of accountability was transferred to this dimension
Equity	1	Chooses fair and equitable criteria for patient care	Approved by experts
	2	In an effort to eliminate discrimination in health care, it practices justice in the health care delivery system	Approved by experts
	3	Respects the rights, individuality and diversity of opinions of colleagues and students	Approved by experts
	4	Follows health and patient care of different ethnicities with equal respect	According to experts, belongs in this dimension
	5	Understands and accepts the diverse nature of research topics and / or patients	Removed from this dimension
	6	Respects these differences in their interactions with others	Removed from this dimension
	7	Respects the rights, individuality and diversity of opinions of colleagues and students	Removed from this dimension
	8	Respects justice in the division of interests and responsibilities	According to experts, belongs in this dimension
Honor And Honesty	1	Provides actions and information in an honest manner	Approved by experts
	2	Develops patient / physician relationships in a way that does not involve financial exploitation, abuse of privacy or sexual harassment	Approved by experts
	3	Reports data regularly, accurately and honestly	Approved by experts
	4	Refuses to violate the principles of professional and personal behavior	Approved by experts
	5	Accepts individual responsibility for decisions related to patient’s care	From the dimension of accountability was transferred to this dimension
	6	Acts dutifully to make arrangements and meet requirements	From the dimension of accountability was transferred to this dimension
	7	Supports scientific standards and makes decisions based on scientific experiments and evidence	removed
	8	Puts more values on the interest of the patient or the subject of the research than his own benefit	removed
	9	Refuses to violate the principles of professional and personal behavior even under conditions of coercion and bribery	Edited grammatically
Altruism	1	Shows self-compassion	Approved by experts
	2	Expresses empathy	Approved by experts
	3	And uses its skills and expertise voluntarily for the betterment of society	Approved by experts
	4	Puts more values on the interest of the patient than his own benefit	it was transferred to this dimension from honor and honesty dimension
	5	Takes the initiative to help in local, national and global crises	According to experts, belongs in this dimension
	6	Helps reduce interpersonal conflicts and seizures	The second round was added to this dimension with the opinion of experts
	7	Collaborates as much as possible in social and charitable activities related to their specialty	The second round was added to this dimension with the opinion of experts
Duty	1	does not seek to advance their professional position at the cost of another person's professional position	Approved by experts
	2	Reports research or medical errors	Approved by experts
	3	Reveals conflict of interest during professional activities and tasks	Approved by experts
	4	Accepts the patient's autonomy and helps him or her make informed decisions	Approved by experts
	5	Acts in a way that demonstrates a commitment to confidentiality	Approved by experts
	6	Participates in correcting those who do not behave professionally	Edited grammatically
	7	Protects patients' privacy	In the second round, it was added with the opinion of experts
Respect	1	Avoids offensive language that contains harsh comments and unfair criticism of others	Approved by experts
	2	Respects the skills and expertise of other members of the treatment team	According to experts, belongs in this dimension
	3	treats people with individual, racial, linguistic and cultural differences politely and forgivingly	According to experts, belongs in this dimension
	4	Maintains your professional grooming in a way that is respected by others	In the first stage, it was removed from this dimension, but in the second stage, it re-entered this dimension with the opinion of experts
	5	Understands and accepts the diverse nature of patients and respects these differences in their interactions with others	From equity dimension was transferred to this dimension
	6	Respects the rights, individuality and diversity of opinions of colleagues and students	From equity dimension was transferred to this dimension

**Table 2:** One-sample t-test of clinical professors' attitudes about the dimensions of professionalism

Variable	number of samples	median	average	standard deviation	value of T	Degrees of freedom	*p*-value
Accountability	82	3	4.25	0.56	20.39	81	0.001
Enrichment	82	3	3.93	0.63	13.3	81	0.001
Equity	82	3	4.2	0.6	18.18	81	0.001
Honor And Honesty	82	3	4.4	0.58	21.95	81	0.001
Altruism	82	3	3.91	0.66	12.5	81	0.001
Duty	82	3	3.98	0.67	13.33	81	0.001
Respect	82	3	4.38	0.51	24.81	81	0.001
Professionalism	82	3	4.14	0.51	20.02	81	0.001

According to the above table, the average score of clinical professors’ attitudes about the dimensions and components of professionalism is significantly higher than the median score of this item.

These results mean that the average dimensions of professionalism are above the median value. According to the average values, among the dimensions professionalism, the highest average was linked to the dimension of honor and honesty with an average of 4.4 and the lowest average was linked to the altruism dimension with an average of 3.91.

**Fig. 1:**
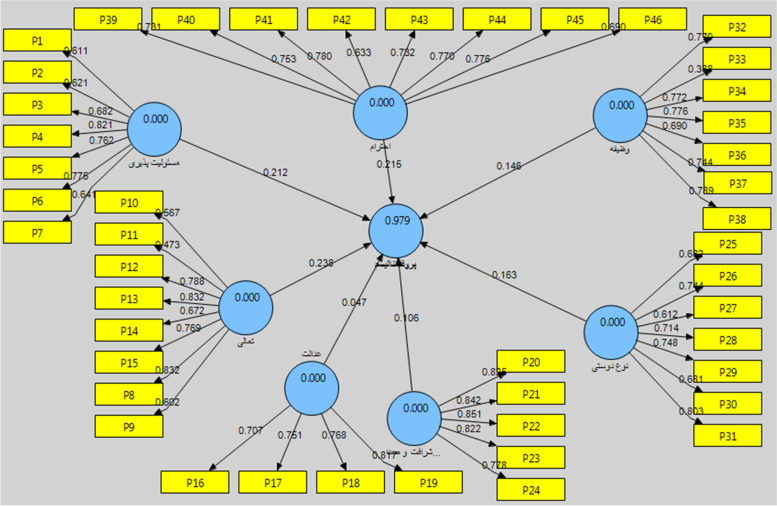
Intensity values of the relationship between research variables in the final model

**Fig. 2:**
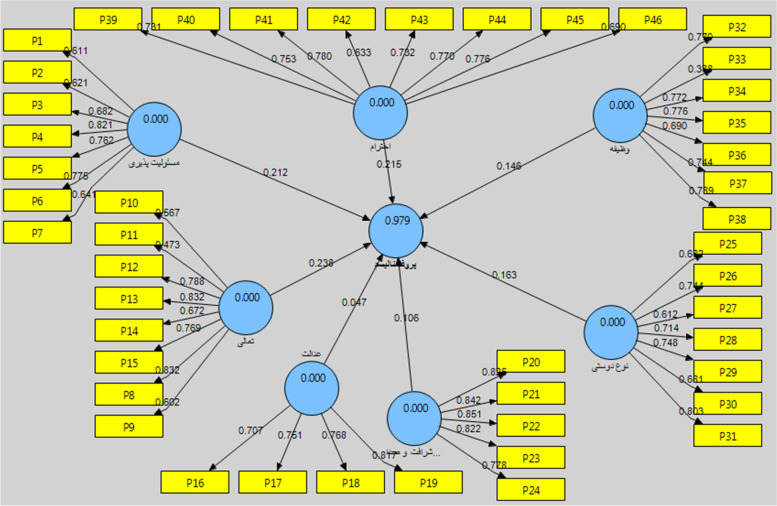
T values of the relationships between the research variables in the final model

Analysis using PLS software has been conducted to investigate the station of different dimensions of medical professionalism in the professional character evaluation instrument with the help of factor analysis. The research model for examining the relationship of different variables is as Fig. 1, and The level of significance of the relations is presented in the Fig. 2:

Other research models values can be seen in the Table 3.

**Table 3:** Review of research hypotheses

Relationships of concepts with indicators in the model		Impact intensity		value of T		Significant level	Result
The dimension of accountability has the ability to measure the variable of professionalism	0.212		11.315		*P* < 0.01		Confirmation of the hypothesis
The dimension of enrichment has the ability to measure the variable of professionalism	0.238		11.78		*P* < 0.01		Confirmation of the hypothesis
The dimension of equity has the ability to measure the variable of professionalism	0.047		3.122		*P* < 0.01		Confirmation of the hypothesis
The dimension of honor and honesty has the ability to measure the variable of professionalism	0.106		4.981		*P* < 0.01		Confirmation of the hypothesis
The dimension of altruism has the ability to measure the variable of professionalism	0.163		9.681		*P* < 0.01		Confirmation of the hypothesis
The dimension of duty has the ability to measure the variable of professionalism	0.146		6.628		*P* < 0.01		Confirmation of the hypothesis
The dimension of respect has the ability to measure the variable of professionalism	0.215		13.519		*P* < 0.01		Confirmation of the hypothesis

From this section we learn that all the dimension of the professionalism variable have the ability to measure this variable.

Measurement model test.

In order to test the measurement model in this study, Cronbach’s alpha and combined reliability were used to evaluate the reliability of the measurement model and convergent validity test and divergent validity were used to evaluate the validity test of the measurement model. The results are shown in Table 4:

**Table 4:** Assessment of indexes of fit of research variables

variable	Cronbach's alpha	Composite reliability	Convergent validity	**CV_red**	**CV_Com**	Status
respect	0.877	0.903	0.54	–-	0.399	Acceptable
enrichment	0.848	0.883	0.494	–-	0.363	Acceptable
honor and honesty	0.882	0.914	0.679	–-	0.508	Acceptable
equity	0.758	0.847	0.58	–-	0.302	Acceptable
accountability	0.83	0.873	0.5	–-	0.338	Acceptable
altruism	0.838	0.879	0.51	–-	0.357	Acceptable
Duty	0.835	0.877	0.514	–-	0.364	Acceptable
Professionalism	0.935	0.943	0.515	0.383	0.353	Acceptable

For the Cronbach’s alpha index, since all values are above 0.7, the Cronbach’s alpha of the research variables is confirmed. Also, the obtained Coefficient of determination was equal to 0.979, which indicates the appropriateness of the ability to measure the variable by the identification components. Finally, the GOF index is 0.728, indicating that the overall fit level of the model is excellent and acceptable.

## Discussion

Six of the identified items have semantic similarity with the original items and only the dimension of justice was identified as a new dimension in this study. To determine experts’ attitude towards different dimensions of medical science professionalism, the components of the evaluation of attitude towards professionalism were reviewed and completed with the help of a survey method. According to research’s experts, the initial components of a questionnaire designed at the University of Pennsylvania, which included the dimension of accountability, enrichment, equity, respect and originality, altruism, duty and respect, were surveyed in the two rounds of surveying, they changed to the components of responsibility, enrichment, justice, honor and honesty, altruism, duty and respect.

In this study, among the initial dimension designed at the University of Pennsylvania, the components of excellence were switched to enrichment, fairness to equity, respect, and nobility changed to honor and honesty, while in Blackall et al.’s (2007) study, only one new componentquity has been added to the basic professionalism dimensions of the American Board of Internal Medicine, which included altruism, accountability, enrichment, duty, honor and honesty, and respect for others [6]. The average of professionalism dimension according to clinical professors, showed that the highest value was given to the dimension of honor and honesty and other dimensions, including respect, accountability, equity, duty, enrichment and altruism respectively .

Joshua Jauregui’s (2016) study entitled Emergency Medicine Resident Perceptions of Medical Professionalism showed that the lowest score is in **altruism** and duty and service and the highest in respect for others [8]. In the current study, the dimension of altruism was in the ranked last similar to the results of Jauregui’s (2016) and Agha Mohammadi’s (2019) research [10].

Research of agha Mohammadi et al. (2017) by examining the attitudes of clinical faculty physicians towards professionalism, found that the professional attitude of medical professors in the indicators of enrichment, honor and honesty and **respect** are at the highest level. However, in the current study, the dimension of honor and honesty has the highest average rank and the dimension of **respect** has the second highest rank, which is different from the results of Jauregui (2016) and Mohammadi in terms of ranking.

A study by Campbell et al. (2007) On 3,504 physicians specializing in six disciplines, including cardiovascular disease, surgery, anesthesia, pediatrics, internal medicine, and family physicians, found that the highest average was given to the **honesty** dimension [9] In the current study, the component of honor and honesty is ranked first, the highest mean was related to the component of honor and honesty, which is similar to the results of Campbell et al. (2007), and in the study of Mohammadi et al. (2019) the honor is in the second rank, which is relatively similar to the findings of this study.

In the current study, the dimension of **enrichment** has the lowest rank, but Agha Mohammadi et al. (2019) ‘s findings in the case of the lowest ranked dimension are different to the results of the current study, and enrichment is in the first rank. In the current study, the dimension of enrichment is similarly in the first rank. The results of Agha Mohammadi et al. (2017) showed that the professional attitude of medical professors in the indicators of enrichment, honor and honesty and respect are at the highest level and respectively in the indicators related to altruism and duty are at the lowest level,the dimension of enrichment was in the first rank [10]

However, the dimension of **equity** in this study has the lowest rank, but in Jauregui’s and Agha mohammadi’s study equity doesn’t have the lowest rank.

In Agha Mohammadi et al. research indicators related to altruism and **duty** are at the lowest level [10]. However, in the current study, it is not at lowest level. The results of Jauregui’s (2016) study entitled emergency medical residents’ perception professionalism showed that the lowest score is in altruism and duty and service and the highest is in respect for others [8].

In this research, **respect** for others is in the second rank and altruism and duty are in the fourth and fifth ranks, and the last rank is assigned to the dimension of equity. Therefore, the findings of this study are different from Jauregui’s research in terms of dimension ranking

The findings of this study can help to localize and validate the instruments for assessing attitudes toward professionalism based on the opinions of clinical professors of Iranian universities of medical sciences and to expand our knowledge in this field. The results of the current study can also pave the way for newer research to expand upon the knowledge of evaluating professionalism based on the views of clinical professors. At the practical level, the findings of this study can be used to develop educational programs and interventions in relevant organizations to promote professional attitudes, and clinical counselors and psychologists can also use the results of this study in the same sense.

According to the results of this study and the opinions of clinical professors and experts of Iranian universities of medical sciences, it is necessary to study Localization and validation of a survey instrument to assess attitudes toward professionalism based on the opinions of clinical professors of Iranian universities of medical sciences. According to the applied results of this study, it is suggested that in the next research, the attitudes of clinical professors towards professionalism in different fields and specialties as well as in different cities and ethnicities be studied and compared. The same research on larger samples should also be done. In this study, the professional attitudes of clinical professors and experts on different dimensions and components of medical professionalism in the professional character evaluation instrument was examined. Other assessment instruments, such as the opinions of students, residents, and even patients, can complement this study.

According to findings, clinical professors have more patient care responsibilities, as a result of increasing responsibility, patient management is inversely related to enrichment and altruism and is directly related to the degree of honor and honesty. In general, education and models for medical professionalism should be formed with more emphasis on the subject of honor and honesty. In order to determine the position of dimensions of medical professionalism in the professionalism evaluation instrument with the help of factor analysis and expert group, the results showed that the enrichment dimension has the highest ability to measure the professionalism variable. In the next ranks, respectively we have, the dimensions of respect, accountability, altruism, duty, honor and honesty and equity. Findings showed that none of the questions of the initial questionnaire needed to be removed and the initial model was acceptable and approved. On the other hand, the fit indices of the model were all within an acceptable range and confirmed the fit and standardization of the model, it should be noted, that because surveying the experts, the proposed reforms were done in several stages, the fit was good in this stage.

## Limitations

Characteristics and the spirituality of participants could be effective on responses, and we didn’t have any control on them. Therefore, Delphi was done in two rounds to confirm answers. Questionnaires were anonymous, but it was possible that the participants’ answers still had a desire for the norm and the answers estimated the people’s attitudes better than the real ones. In other words, people tend to declare social ideals as their attitude. Participants were busy and answering the questionnaires was time-consuming. For this limitation, we selected interested experts to assessing Medical Professionalism and explained the necessity of this research. The lack of questionnaire questions makes it impossible to make accurate judgments about the superiority of attitude in one area over other areas.

## Conclusion

According to the results of this study, the most dimensions in PSCOM are accepted by experts and teachers, but there are many differences in details. If we want to have an accurate tool for measuring attitudes toward professionalism, then it is necessary to study localization and validation of an instrument to assess attitudes toward professionalism in every new community. Delphi and factor analysis could be useful for assessing tool in a new community. It is suggested that in the next research, the attitudes of clinical professors towards professionalism in different fields and specialties as well as in different communities be studied and compared.

## Data Availability

The datasets used and analyzed during the current study are not publically available due to ethical restriction and personal data protections but are available from the corresponding author on reasonable request.
